# How Self-Belief in Creativity and Well-Being Is Associated with Life Satisfaction, Meaning in Life, and Psychological Richness: The Mediating Effect of Creative Self-Efficacy

**DOI:** 10.3390/jintelligence13030025

**Published:** 2025-02-20

**Authors:** Dongdong Liu, Chenggang Wu, Yaxuan Meng, Jing Dang

**Affiliations:** 1 College of Teacher Education, Suqian University, Suqian 223800, China; 23179@squ.edu.cn; 2 Key Laboratory of Multilingual Education with AI, School of Education, Shanghai International Studies University, Shanghai 202620, China; 3 Institute of Language Sciences, Shanghai International Studies University, Shanghai 202620, China; 4 School of Foreign Studies, Shanghai University of Finance and Economics, Shanghai 200433, China; mengyaxuan@mail.shufe.edu.cn; 5 College of Education, Inner Mongolia Normal University, Hohhot 010010, China; 20200028@imnu.edu.cn

**Keywords:** self-beliefs in creativity and well-being, life satisfaction, psychological richness, creative self-efficacy

## Abstract

This study aimed to validate the Chinese version of the self-beliefs in creativity and well-being (SBCWs) and probe its associations with life satisfaction, meaning in life, and psychological richness. Additionally, it explored the mediating role of creative self-efficacy between SBCWs and well-being. Evidence of the reliability and validity of the Chinese SBCWs were provided, with their correlation to well-being measures offering further validity support. Mediation analyses showed that creative self-efficacy mediated the links between SBCW and life satisfaction, psychological richness, and presence of meaning, but not between SBCW and the search for meaning. Differential SBCW predictions for short- and long-term well-being were confirmed, suggesting SBCW’s split of short-term and long-term focus. The study highlights creativity and creative self-efficacy’s importance in well-being.

## 1. Introduction

It is abundantly clear that subjective well-being has been an undercurrent and consistent academic pursuit for decades. This is due to the fact that subjective well-being has numerous beneficial impacts on individuals’ lives. Happiness in life appears to be a universal goal across nations (Eid and Larsen 2008). However, psychological well-being is more than happiness, also containing other aspects such as self-acceptance, purpose in life, positive relations with others, and personal growth (Ryff and Keyes 1995). Although subjective well-being and psychological well-being have slightly different components for constructing a good life, both of them affirm that well-being is closely associated with numerous successful and healthy outcomes. These include organizational citizenship behaviors, job performance, income, material wealth, instrumental support, satisfaction with friends, marital happiness, global health, optimism, social activity, competence, partner-rated attraction, and many others (Lyubomirsky et al. 2005). The Character Strength and Virtues Model, which includes six virtues—courage, humanity, justice, temperance, transcendence, wisdom, and knowledge—was put forth by Peterson and Seligman (2004). From this model, creativity is a key component of intelligence and knowledge. According to the theories of personal (Runco 2007) and little-c (Kaufman and Beghetto 2009) creativity, creativity constitutes an essential part of well-being. For instance, when an individual needs to resolve a conflict between two close friends, demonstrating creativity is beneficial for both the interpersonal relationship and overall well-being. Hence, the relationship between creativity and well-being is inherently linked, and this will be introduced in the next section.

### 1.1. Creativity and Well-Being

A recent meta-analysis (Acar et al. 2021) identified a modest yet meaningful positive correlation between creativity and well-being, with a correlation coefficient of 0.14. This significant finding has been corroborated and replicated across diverse groups. Creative professionals (Smith et al. 2022), employees in the hospitality sector (Mashkoor and Muhammad 2024), those in the chemical, high-tech, and consumer products industries (Amabile et al. 2005), and college students (Li and Wu 2024) have all contributed to validating this relationship. Baas et al. (2008) conducted a meta-analysis to investigate the connection between moods and creativity, determining that creativity can be augmented by positive moods characterized by high arousal and associated with approach motivation. There is a growing body of evidence substantiating the facilitative impact of positive affect on creativity (Silvia et al. 2014), particularly in the context of everyday creativity within daily life. As posited by Richards (2018), everyday creativity holds a pivotal position in psychological development. Such activities can foster the formation of interpersonal relationships, the generation of novel ideas, and the enhancement of competence, all of which collectively establish a novel foundation for general well-being (Jean-Berluche 2024).

This relationship became even more prominent during the COVID-19 pandemic. For instance, Fiori et al. (2022) demonstrated that creativity was linked to well-being, and more-creative individuals exhibited greater life satisfaction, even after controlling for perceived stress. Mediation analyses also indicated that creativity nurtured positive affects and led to enhanced positive COVID-19 experiences. In a same vein, Tang et al. (2021) provided cross-cultural evidence in favor of using creativity to improve well-being throughout the epidemic. Individuals from China, Germany, and the United States exhibited a positive association between creative process engagement and self-reported creative growth, which was also correlated with flourishing well-being. Collectively, these studies firmly establish the positive influence of creativity on well-being, particularly in times of hardship.

The association between creativity and well-being has also been observed across various age cohorts, including teenagers (González Moreno and Molero Jurado 2023) and aging adults (Turiano et al. 2012). Turiano et al. (2012) discovered that creativity served as a predictor of mortality risk, where an elevation in creativity corresponded to a substantial decrease in the likelihood of mortality. A more recent study (González Moreno and Molero Jurado 2023) also established a positive correlation between creativity and self-esteem, a favorable personality trait that bears relevance to academic performance (Yu et al. 2022). Beyond self-reported outcomes, Bolwerk et al. (2014) put forward neurological evidence. Following a 10-week art intervention that encompassed extensive creative art activities, the intervention group demonstrated a more significant spatial improvement in the functional connectivity between the posterior cingulated cortex (PCC) and the frontal and parietal cortices when compared to the control group. Significantly, this functional connectivity was also intertwined with psychological resilience. These findings support the hypothesis that engaging in creative artistic activities can modify the default mode network pattern, and such a modification might potentially be linked to psychological well-being. Similarly, Tan et al. (2021) conducted two experiments to examine how a creativity priming task improved self-reported subjective well-being in addition to providing correlational evidence supporting the positive relationship between creativity and subjective well-being while accounting for self-perceived stress. These experiments revealed that individuals who underwent a creativity priming exhibited a more pronounced increase in subjective well-being compared to the control group. Based on the preceding studies, it becomes patently evident that creativity serves as a potent enhancer for both psychological well-being and subjective well-being.

### 1.2. Self-Beliefs in Creativity and Well-Being and Creative Self-Efficacy

In light of the firmly established nexus between creativity and well-being, Holinger and Kaufman (2024) recently proffered a novel assessment to gauge self-beliefs in creativity and well-being (SBCWs) and discerned that such self-beliefs manifested a positive correlation with creative self-efficacy. Owing to the dearth of an extant measurement instrument bridging creativity and well-being, Holinger and Kaufman (2024) partitioned the items that correlated creativity with well-being into short-term and long-term classifications (SBCW-S and SBCW-L). Precisely, short-term well-being was tethered to hedonia, whereas long-term well-being was affiliated with eudaimonia. The former (hedonia) construes well-being as the presence of positivity and the attenuation of negativity, yet the latter (eudaimonia) conceives well-being as the actualization of one’s latent potentials (Deci and Ryan 2008). Grounded in content validity scrutiny, Holinger and Kaufman (2024) initially retained 17 items and, subsequent to an exploratory factor analysis (EFA), 15 items were conserved, with 7 items appraising short-term well-being and 8 items evaluating long-term well-being. Anchored on the EFA outcomes, Holinger and Kaufman (2024) further furnished confirmatory factor analysis results as substantiation of construct validity. The overarching two-factor model was corroborated, with a satisfactory model fit. Significantly, Holinger and Kaufman (2024) also demonstrated that self-beliefs in creativity and well-being (SBCWs) were positively correlated with subjective well-being, and intriguingly, long-term well-being (SBCW-L) rather than short-term well-being (SBCW-S) was intertwined with life satisfaction. These findings further authenticated the demarcation between self-belief in creativity and long-term and short-term well-being, intimating the exigency of discriminating between the two varieties of well-being.

Furthermore, Holinger and Kaufman (2024) likewise discovered a correlation between SBCW and creative self-efficacy. Creative self-efficacy is defined as the “conviction that one possesses the capacity to generate creative outcomes” (Tierney and Farmer 2002). Evidently, this concept posits a connection between creative self-efficacy and the production of products, which is emphasized within corporate settings. Beghetto’s work (Beghetto 2006) extended the concept of creative self-efficacy to the educational realm, revealing that students with high levels of creative self-efficacy also exhibited relatively elevated positive beliefs regarding their academic capabilities and even manifested an augmented intention to pursue higher education and engage in after-school academic and group undertakings. These findings signify a positive correlation between creative self-efficacy and favorable academic dispositions, and these results were further replicated by high school students from Iran (Kashanian and Sheikhpour 2024). In addition to students, Cayirdag (2017) demonstrated that teachers’ creative self-efficacy predicts their creativity-fostering behaviors, suggesting that teachers can assume a pivotal role in nurturing students’ creativity and that this role may potentially be augmented by creative self-efficacy. In another meta-analysis of creative self-efficacy and creativity assessments, Haase et al. (2018) ascertained that creative self-efficacy is positively associated with self-reported creativity, the creative process, and creative products, although the strength of the relationship is most pronounced between creative self-efficacy and self-reported creativity. These investigations uniformly corroborate a beneficial function of creative self-efficacy, and this advantageous role can also be extrapolated to SBCW, as manifested by the positive correlation between creative self-efficacy and SBCW. Nevertheless, as contended by Holinger and Kaufman (2024), it remains incumbent upon future research to explore the manner in which SBCW and creative self-efficacy are related to other well-being consequences and how SBCW could be measured in non-Western countries, such as China. Conceivably, SBCW could be positively associated with well-being outcomes through the mediating effect of creative self-efficacy, since a personalized sense of creative self-efficacy can impact positive self-perceptions of creativity and well-being. This supposition will be scrutinized in the current study. Prior to outlining the present study, it is essential to further explicate the concept of a good life and incorporate a hitherto overlooked yet significant perspective, namely, the psychologically rich perspective.

### 1.3. What Is a Good Life? A Happy Life, a Meaningful Life, or a Psychologically Rich Life?

The delineation of a good life is intricate and multifarious. As expounded in the preceding section, a good life can be defined as either a happy life, which is typified by a state of contentment, or a meaningful life, which is characterized by a state of coherence, purposiveness, and comprehensiveness (King and Hicks 2021). Moreover, Steger et al. (2006) posited that the measurement of meaning in life encompasses both the presence and the search for meaning. Searching for meaning has been found to be associated with negative affectivities, such as fear and depression (Steger et al. 2006), and searching for meaning does not necessarily lead to the presence of meaning (Steger et al. 2008). In a similar vein, Li et al. (2021) carried out a meta-analysis with the aim of exploring the relationships among the presence of meaning, the search for meaning, and subjective well-being. Their findings indicated that the effect size of the relationship between the presence of meaning and meaning in life was moderate. However, the effect size of the relationship between the search for meaning and well-being was relatively small (Li et al. 2021). Nevertheless, conflicting evidence has emerged, indicating that within individuals, the daily pursuit of meaning was positively correlated with the presence of meaning and well-being. Lagged analyses have also substantiated such a salutary impact of the search for meaning on the presence of meaning (Newman et al. 2018). These outcomes imply that the search for meaning could also serve as a propitious catalyst for well-being. Consequently, the present study will appraise both the presence and the search for meaning. Kaufman (2018) contended that creativity and meaning in life are intrinsically intertwined, and elaborated a temporal model encompassing past, present, and future pathways to illustrate how creativity can augment meaning (Kaufman 2018). More precisely, creativity enables individuals to seek coherence by delving into their past experiences. In the present moment, it serves to facilitate people’s mood regulation, thereby endowing their lives with significance. Looking ahead to the future, creativity equips people to confront life by grasping the purpose of meaning-making through leaving legacies. Creativity and meaning are mutual facilitators, particularly in an era beset by existential perils, such as natural disasters (Kaufman 2025).

Beyond a happy and a meaningful life, one could also be dedicated to a psychologically rich life, even at the cost of foregoing a happy or a meaningful life (Oishi and Westgate 2021). A psychologically rich life is denoted by “a multiplicity of captivating and perspective-altering experiences” (Oishi and Westgate 2021). Indeed, a segment of the population (7–17%) gravitates towards a psychologically rich life, and certain Americans (28%) and Koreans (35%) have professed that they harbored regrets and would have preferred to lead a more psychologically rich life (Oishi et al. 2020). Oishi et al. (2021) further demonstrated that students who scored highly in psychological richness were more inclined to study abroad and undertake short excursions (Oishi et al. 2021). Such a positive correlation was not discernible for life satisfaction, positive affect, and meaning in life. These findings further accentuate the distinctions between a happy life, a meaningful life, and a psychologically rich life. In order to probe how a psychologically rich life is related to other individual variances, Oishi et al. (2019) devised a questionnaire to gauge psychological richness and ascertained that psychological richness was prognosticated by openness to experience and extraversion. This discovery is captivating as it intimates a positive nexus between psychological richness and creativity, given the burgeoning evidence corroborating a positive association between openness to experience and creativity (McCrae 1987). For example, Tan et al. (2019) discovered that openness to experience augmented creativity via intrinsic motivation and creative process engagement (Tan et al. 2019). Analogously, leading a psychologically rich life entails a panoply of experiences and perpetual perspective metamorphoses, which are intrinsically linked to openness to experience. Hence, it is tenable to posit that self-beliefs in creativity and well-being are correlated with another frequently overlooked dimension of well-being, namely psychological richness.

### 1.4. The Present Study

Based on this comprehensive and in-depth analyses of the existing literature, several unresolved questions have come to light. Firstly, although the SBCW questionnaire has been developed and its validity established within one Western country, its applicability and validity in other cultural contexts remain ambiguous. Secondly, as argued by Oishi et al. (2021), a good life might include not only a happy and meaningful life but also a psychologically rich life, whereas Holinger and Kaufman (2024) only examined the link between SBCW and subjective happiness and life satisfaction. A psychologically rich life intrinsically involves creative activities and the exploration of novel experiences. Consequently, it is of significant value to investigate how SBCW is related to psychological richness. Thirdly, although a connection has been identified between creative self-efficacy and SBCW, the manner in which creative self-efficacy might mediate the relationship between SBCW and well-being remains unclear.

The present study endeavors to address the above three as-yet-unanswered research questions. Specifically, the present study posits the following research questions:

**RQ1:** *Can the SBCW questionnaire be validated within a Chinese sample?*
**RQ2:** *What is the relationship between SBCW and life satisfaction, the presence of meaning, the search for meaning, and psychological richness?*
**RQ3:** *How does creative self-efficacy mediate the relationship between SBCW and life satisfaction, the presence of meaning, the search for meaning, and psychological richness?*

In light of the aforementioned research inquiries, pertinent hypotheses can be posited, rooted in the prior literature and theoretical frameworks. It is anticipated that the SBCW questionnaire is correlated with diverse dimensions of well-being, such as life satisfaction (Holinger and Kaufman 2024), the presence of meaning, the search for meaning, and psychological richness. For the purpose of facilitating the positive impact of SBCW on well-being, creative self-efficacy is requisite. Grounded in the positive association between SBCW and creative self-efficacy (Holinger and Kaufman 2024), it is hypothesized that creative self-efficacy may serve as a mediator in the relationship between SBCW and well-being.

## 2. Methods

### 2.1. Participants

A cohort of Chinese young adults, consisting of 434 individuals (comprising 73 male students), with a mean age of 20.20 years, completed a hybrid questionnaire. The vast majority of the participants were undergraduate students (numbering 431, while only 3 were postgraduate students). Among the undergraduate students, the majority were sophomores (183) and juniors (200). In contrast, there were relatively small numbers of freshmen (21) and seniors (27). The majority of the participants were from Jiangsu (359), and the rest of the participants were from Jiangxi (58), Inner Mongolia (10), Guizhou (3), Shanghai (1), Shanxi (1), and Hubei (2). All participants provided a consent form prior to commencing the filling out of the questionnaire. Additionally, they were permitted to withdraw from the participation at any time.

### 2.2. Measurements, Procedure, and Data Analysis

In addition to the initial segment collecting demographic information, the questionnaire was composed of several sections that measured various aspects such as life satisfaction, meaning, psychological richness, self-beliefs of creativity and well-being, and creative self-efficacy on a 7-point scale, ranging from 1 (strongly disagree) to 7 (strongly agree). Specifically, life satisfaction was gauged by The Satisfaction With Life Scale (Diener et al. 1985). This scale comprises five items and evaluates the participants’ subjective life satisfaction, possessing adequate reliability and validity evidence (Cronbach’s α = 0.88, CFI = 1.00, GFI = 1.00, RMSEA = 0.04, SRMR = 0.01). Meaning was measured by the meaning in life questionnaire (Steger et al. 2006). This scale contains 10 items, with 5 items measuring the presence of meaning in life and five items measuring the search for meaning in life (Cronbach’s α = 0.70, CFI = 1.00, GFI = 1.00, RMSEA = 0.08, SRMR = 0.03 for presence; Cronbach’s α = 0.94, CFI = 1.00, GFI = 1.00, RMSEA = 0.08, SRMR = 0.03 for search). Psychological richness (Oishi et al. 2019) was measured by the psychologically rich life questionnaire, which has 12 items (Cronbach’s α = 0.95, CFI = 1.00, GFI = 1.00, RMSEA = 0.09, SRMR = 0.04). Creative self-efficacy was measured by the methods used in Beghetto’s study (Beghetto 2006), consisting of five items (Cronbach’s α = 0.97, CFI = 1.00, GFI = 1.00, RMSEA = 0.00, SRMR = 0.02). Self-beliefs in creativity and well-being were measured using Holinger and Kaufman’s methods (Holinger and Kaufman 2024). The scale (Cronbach’s α = 0.97, CFI = 1.00, GFI = 1.00, RMSEA = 0.08, SRMR = 0.05) consisted of seven items measuring short-term well-being (hedonia) and eight items measuring long-term well-being (eudaimonia). The questionnaire was translated by the authors themselves. Subsequently, a back-translation process was carried out in collaboration with a postgraduate student whose major was education. The final version of the questionnaire was determined based on the consensus reached among those involved in the process.

The questionnaire was distributed to undergraduates via various WeChat groups associated with course work, with the assistance of the colleagues and friends of the two authors. As soon as the participants opened the link, they would begin filling out the questionnaire, provided they gave their informed consent. On average, the participants took roughly 10 min to complete the questionnaire.

Regarding data analysis, we utilized JASP version 0.18 (Love et al. 2019) to perform descriptive statistical analyses, correlational analyses, and mediation analyses. The mediation analyses were carried out through the Structural Equation Modeling (SEM) function in JASP, employing the standard method. In these analyses, the short- and long-form measures of SBCW served as predictors, creative self-efficacy functioned as the mediator, and well-being was designated as the outcome variable.

## 3. Results

Table 1 presents the descriptive statistics and correlation results of the related variables. It is evident that the participants demonstrated a relatively medium to high positive attitude towards their lives, particularly in terms of having a psychologically rich life and a meaningful life, with all values being larger than 4.0/7.0. Moreover, the participants also exhibited a relatively positive attitude towards creativity and well-being, and they also showed relatively high creative self-efficacy, all being higher than 4.0.

From the correlation results (Table 1), it is also evident that all of the variables were closely correlated (all *rs* > 0.11). This suggests that a good life encompasses all three aspects. Moreover, creativity and well-being are also related to creative self-efficacy as well as the different perspectives of a good life. Specifically, SBCW-short and -long were positively related to creative self-efficacy (*r* = 0.73 for short, *r* = 0.74 for long), satisfaction (*r* = 0.32 for short, *r* = 0.43 for long), psychological richness (*r* = 0.50 for short, *r* = 0.57 for long), the presence of meaning (*r* = 0.47 for short, *r* = 0.59 for long), and the search for meaning (*r* = 0.50 for short, *r* = 0.44 for long).

**Figure 1:**
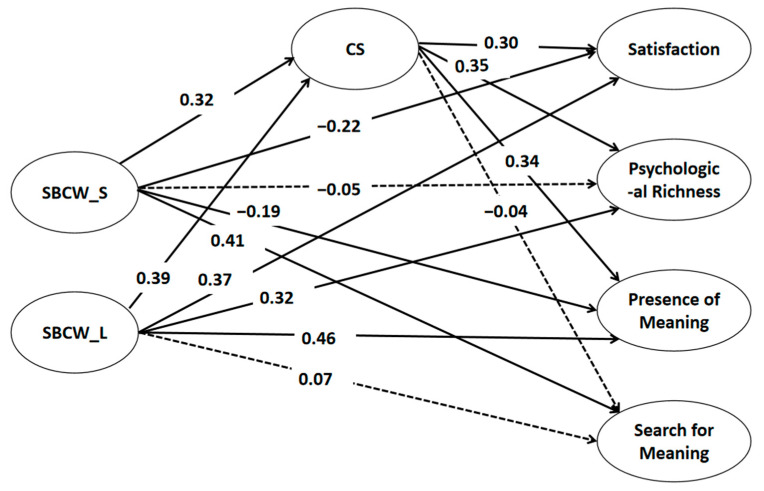
The model results. Note. All *p* values are less than 0.01. The dashed lines represent relationships that are non-significant. SBCW_S: self-belief in creativity and well-being in the short term; SBCW_L: self-belief in creativity and well-being in the long term; CS: creative self-efficacy.

The mediation analysis revealed that SBCW for the short term positively predicted searching for meaning. In contrast, SBCW for the long term could directly predict life satisfaction, presence of meaning, and psychologically rich life. Creative self-efficacy (CS) mediated the relationship between SBCW (both long and short term) and life satisfaction, a psychologically rich life, and meaning presence. However, the mediation effect was not found for the relationship between SBCW and searching for meaning in life. This suggests that different aspects of SBCW have distinct relationships with various aspects of life satisfaction and meaning, and that creative self-efficacy plays a specific role in mediating some of these relationships but not others (see Table 2 and Figure 1 for more details).

## 4. Discussion

In addition to validating a Chinese version of the SBCW questionnaire, the primary goal of the present study was to explore how SBCW was related to different aspects of well-being—life satisfaction, meaning, and psychological richness—and examine how the relationship between SBCW and well-being was mediated by creative self-efficacy. Overall, the present study confirmed the structure of the SBCW questionnaire in a Chinese sample, and the correlation between SBCW and other well-being measurements further provide criterion-related validity. More importantly, the present study showed that SBCW for short-term well-being and long-term well-being had different relationship with life satisfaction, the presence of meaning, the search for meaning, and psychological richness. The mediating effect of creative self-efficacy on the association between SBCW and different aspects of well-being was also varied.

### 4.1. Consistent Positive Direct Effect of SBCW for Long-Term Well-Being on Life Satisfaction, Psychological Richness, and Presence of Meaning

In line with the study of Holinger and Kaufman (2024), we found that SBCW for long-term well-being showed a direct impact on life satisfaction. In addition, SBCW for long-term well-being also directly impacted the presence of meaning. From the theorized content of the SBCW questionnaire for long-term well-being, it also captures meaning, the purpose of life, positive personal relationships, and personal growth, all of which are clearly related to presence of meaning (King and Hicks 2021). This content and criterion-related validity were further strengthened by the finding of the direct prediction via SBCW for long-term well-being of the presence of meaning. More importantly, we also observed a positive direct impact of SBCW for long-term well-being on psychological richness. Although, from the items of the SBCW questionnaire for long-term well-being, Holinger and Kaufman did not consider psychological richness, based on the inherent intricate relationship between creativity and a psychologically rich life, it was plausible to find a positive correlation between SBCW for long-term well-being and psychological richness. Therefore, the present study went beyond the previous literature by showcasing that a positive belief in creativity and well-being increases psychological richness, a neglected aspect of well-being and a good life.

It is rather intriguing to observe that the SBCW questionnaire, which was designed for long-term well-being, did not prove capable of predicting the search for meaning. This finding aligns with prior studies showing a negative correlation between the search for meaning and negative affects (Steger et al. 2006). Essentially, the search for meaning appears to bear no relation to long-term well-being, as meaning is not attained through the act of searching but is instead embedded within social connections, cognitive coherence, life purposes, and positive affectivities (King and Hicks 2021). In a similar vein, Khumalo et al. (2014) illustrated that the search for meaning did not mediate the relationship between existential well-being and certain indices of psychological well-being. This further elucidates the reason why the SBCW questionnaire for long-term well-being failed to predict the search for meaning. It implies that if an individual holds the belief that creativity has implications for long-term well-being, there would be relatively little necessity for further pursuit of meaning, given that meaning is already present in such a context.

### 4.2. Direct Influence of SBCW for Short-Term Well-Being on Satisfaction and Meaning

In contrast to the SBCW questionnaire designed for long-term well-being, the SBCW questionnaire formulated for short-term well-being did not manifest a direct influence on psychological richness, and had a negative impact on satisfaction. Nevertheless, the SBCW questionnaire for short-term well-being directly predicted the search for meaning. This finding implies that if an individual holds the belief in a positive connection between creativity and short-term well-being, it is probable that they would embark on the quest for meaning. This extends prior evidence, which demonstrated a negative correlation between the search for meaning and subjective well-being (Cohen and Cairns 2012), by revealing a positive association between short-term well-being and the search for meaning. It is reasonable to assume that individuals experiencing short-term well-being might still aspire to attain long-term well-being and thus have a need for meaning, which would necessitate a further search for meaning. On the other hand, if an individual enjoys high levels of long-term well-being, the presence of meaning in their life is already established. This prediction gains further support from the negative prediction by the SBCW questionnaire of short-term well-being regarding the presence of meaning and satisfaction.

These disparate direct impacts of the SBCW questionnaires for short-term and long-term well-being on life satisfaction, psychological richness, and meaning further corroborate the distinct roles of the SBCW questionnaires for short-term and long-term well-being and the theoretical framework that construes SBCW in terms of two types of well-being. Further investigations into the diverse roles played by the SBCW questionnaires for long-term and short-term well-being in other aspects of mental health, such as anxiety, depression, and eating disorders, are essential.

### 4.3. The Mediating Effect of Creative Self-Efficacy on the Relationship Between SBCW and Well-Being

In addition to its direct impact on life satisfaction, psychological richness, and the presence of meaning, the SBCW questionnaire for long-term well-being also partially mediates the associations between SBCW for long-term well-being and these aspects of well-being. This not only furnishes further validation for the external construct of SBCW but also accentuates the crucial function of creative self-efficacy—defined as a subjective assessment of one’s capacity to generate novel ideas and outcomes (Beghetto 2006)—in augmenting well-being when SBCW is taken into account. The resultant implications are that if individuals are of the conviction that creativity can confer benefits upon well-being, creative self-efficacy will then go on to further enhance diverse facets of well-being, encompassing life satisfaction, psychological richness, and the presence of meaning. Accordingly, the present study extends the positive correlation between SBCW and creative self-efficacy, as initially observed by Holinger and Kaufman (2024), by demonstrating the mediating effect of creative self-efficacy. This also suggests that increasing creative self-efficacy holds significant importance, given that it can fortify the nexus between SBCW and well-being and amplify the contribution of creativity to well-being.

The augmentation of creative self-efficacy garners further substantiation from its full mediating role in the relationship between SBCW for short-term well-being and psychological richness. Despite the fact that SBCW for short-term well-being fails to exhibit a direct impact on psychological richness, creative self-efficacy manages to forge a potential connection between SBCW and well-being. This suggests that creative self-efficacy has the potential to enhance psychological richness, even in scenarios where creativity is only conducive to short-term well-being. It is also plausible that the transition from short-term well-being to long-term well-being, with the aim of elevating psychological richness, necessitates the engagement of creative self-efficacy. Individuals with high levels of creative self-efficacy are inclined to partake in social activities and solidify social bonds (Beghetto 2006), which in turn augment well-being metrics such as life satisfaction. This contention is buttressed by evidence indicating that creative self-efficacy mediates the correlation between the Big Five personality traits and mental well-being (Fino and Sun 2022).

However, it is rather intriguing to note that creative self-efficacy exhibits no mediating effect in relation to the search for meaning. These findings expand upon the research of Han et al. (2023), who discerned that general self-efficacy mediates the relationship between creativity and meaning in life. Nonetheless, Han et al. (2023) did not differentiate between the presence of meaning and the search for meaning, omitted creative self-efficacy from their analysis, and operationalized creativity as the aggregate score of the Kaufman Domains of Creativity Scale (K-DOCS) (Kaufman 2012). Such methodological choices precluded a comprehensive exploration of the disparities between the presence of and search for meaning, as well as the distinctiveness of creative self-efficacy. By treating meaning as comprising both the presence of and search for meaning (Steger et al. 2006), it becomes evident that creative self-efficacy assumes divergent mediating roles in the relationship between SBCW and meaning in life.

### 4.4. Limitations and Future Directions

The present study, notwithstanding its contributions, is not without certain limitations that undeniably call for further in-depth exploration. Firstly, the employment of a cross-sectional design in this study imposes inherent constraints when it comes to unraveling the intricate and dynamic nature, as well as the developmental trajectory, of the SBCW questionnaire and its associations with diverse aspects of well-being. A cross-sectional design merely offers a snapshot of the variables at a specific point in time, precluding a comprehensive understanding of how these constructs evolve and interact over an extended period. In light of this, future research endeavors would be well-advised to adopt a longitudinal design. By doing so, researchers can meticulously track the progression of SBCW over time, discerning the factors that precipitate its development and how it, in turn, forecasts well-being outcomes in a more temporally nuanced manner. Secondly, the current study has adopted a somewhat one-sided perspective by predominantly focusing on the positive dimensions of life. However, life is replete with a spectrum of experiences, and it is essential to consider the negative counterparts as well. Anxiety, social exclusion, and stress are just a few examples of such negative consequences that warrant further scrutiny in relation to SBCW. Understanding how SBCW interfaces with these adverse states can provide a more holistic view of its role within the broader psychological landscape. Thirdly, the sample in the current study was predominantly made up of female college students from Jiangsu, China. These students are specifically young adults. This characteristic significantly restricts the extent to which the study’s findings can be applied to the elderly population, as the present study showed a relatively positive bias on the scales. Consequently, for future research undertakings, it is highly advisable to recruit a substantially larger number of male students. Additionally, a more diverse sample should be assembled by enlisting subjects from a broad spectrum of geographical regions and covering a wide age range, with a particular emphasis on including the elderly. By adopting such a strategic approach, the external validity of the study’s findings will be markedly enhanced.

## Figures and Tables

**Table 1:** Descriptive statistics and correlations of all variables.

	Mean	SD	1	2	3	4	5	6	7
1. Satisfaction	3.97	1.19	___						
2. PR	4.43	1.20	0.65 ***	___					
3. Presence	4.21	0.96	0.66 ***	0.75 ***	___				
4. Searching	4.52	1.23	0.11 *	0.24 ***	0.24 ***	___			
5. CW_short	4.88	1.12	0.32 ***	0.50 ***	0.47 ***	0.50 ***	___		
6. CW_long	4.67	1.12	0.43 ***	0.57 ***	0.59 ***	0.44 ***	0.84 ***	___	
7. CS	4.39	1.17	0.43 ***	0.57 ***	0.56 ***	0.35 ***	0.73 ***	0.74 ***	___

Note. PR: psychological richness; CW_short: self-beliefs in creativity and well-being in the short term; CW_long: self-beliefs in creativity and well-being in the long term; CS: creative self-efficacy; * *p* < 0.05; *** *p* < 0.001.

**Table 2:** Direct effects of belief in creativity and well-being on life satisfaction, presence of meaning, searching for meaning, and psychologically rich life, and the indirect effect of CS.

	Total Effects	Direct Effects	Indirect Effects via CS
	[95% CI]	[95% CI]	[95% CI]
CW_short → satisfaction	−0.12 [−0.27, 0.02]	−0.22 ** [−0.36, −0.08]	0.10 *** [0.05, 0.15]
CW_long → satisfaction	0.49 *** [0.35, 0.63]	0.37 *** [−0.51, −0.16]	0.12 *** [0.06, 0.18]
CW_short → PR	0.06 [−0.07, 0.19]	−0.05 [−0.18, 0.08]	0.11 *** [0.06, 0.16]
CW_long → PR	0.46 *** [0.33, 0.59]	0.32 *** [0.19, 0.45]	0.14 *** [0.08, 0.19]
CW_short → Presence	−0.08 [−0.21, 0.05]	−0.19 ** [−0.32, −0.06]	0.11 *** [0.06, 0.16]
CW_long → Presence	0.59 *** [0.46, 0.72]	0.46 *** [0.33, 0.59]	0.13 *** [0.08, 0.19]
CW_short → Searching	0.39 *** [0.26, 0.53]	0.41 *** [0.26, 0.55]	−0.01 [−0.05, 0.03]
CW_long → Searching	0.06 [−0.08, 0.20]	0.07 [−0.07, 0.22]	−0.01 [−0.06, 0.03]

Note. PR: psychological richness; CW_short: self-beliefs in creativity and well-being in the short term; CW_long: self-beliefs in creativity and well-being in the long term; CS: creative self-efficacy; ** *p* < 0.01; *** *p* < 0.001.

## Data Availability

The data presented in this study are available on request from the corresponding author to protect the confidentiality of the participants.

## References

[B1-jintelligence-13-00025] Acar Selcuk, Tadik Harun, Myers Danielle, Sman Carian Van der, Uysal Recep (2021). Creativity and well-being: A meta-analysis. The Journal of Creative Behavior.

[B2-jintelligence-13-00025] Amabile Teresa M., Barsade Sigal G., Mueller Jennifer S., Staw Barry M. (2005). Affect and creativity at work. Administrative Science Quarterly.

[B3-jintelligence-13-00025] Baas Matthijs, De Dreu Carsten K. W., Nijstad Bernard A. (2008). A Meta-Analysis of 25 Years of Mood–Creativity Research: Hedonic Tone, Activation, or Regulatory Focus?. Psychological Bulletin.

[B4-jintelligence-13-00025] Beghetto Ronald A. (2006). Creative self-efficacy: Correlates in middle and secondary students. Creativity Research Journal.

[B5-jintelligence-13-00025] Bolwerk Anne, Mack-Andrick Jessica, Lang Frieder R., Dörfler Arnd, Maihöfner Christian (2014). How art changes your brain: Differential effects of visual art production and cognitive art evaluation on functional brain connectivity. PLoS ONE.

[B6-jintelligence-13-00025] Cayirdag Nur (2017). Creativity Fostering Teaching: Impact of Creative Self-efficacy and Teacher Efficacy. Kuram ve Uygulamada Egitim Bilimleri.

[B7-jintelligence-13-00025] Cohen Karen, Cairns David (2012). Is searching for meaning in life associated with reduced subjective well-being? Confirmation and possible moderators. Journal of Happiness Studies.

[B8-jintelligence-13-00025] Deci Edward L., Ryan Richard M. (2008). Hedonia, eudaimonia, and well-being: An introduction. Journal of Happiness Studies.

[B9-jintelligence-13-00025] Diener Ed, Emmons Robert A., Larsen Randy J., Griffin Sharon (1985). The satisfaction with life scale. Journal of Personality Assessment.

[B10-jintelligence-13-00025] Eid Michael, Larsen Randy J. (2008). The Science of Subjective Well-Being.

[B11-jintelligence-13-00025] Fino Emanuele, Sun Siyu (2022). ‘Let us create!’: The mediating role of Creative Self-Efficacy between personality and Mental Well-Being in university students. Personality and Individual Differences.

[B12-jintelligence-13-00025] Fiori Marina, Fischer Silke, Barabasch Antje (2022). Creativity is associated with higher well-being and more positive COVID-19 experience. Personality and Individual Differences.

[B13-jintelligence-13-00025] González Moreno Alba, Molero Jurado María del Mar (2023). Creativity as a Positive Factor in the Adolescence Stage: Relations with Academic Performance, Stress and Self-Esteem. Behavioral Sciences.

[B14-jintelligence-13-00025] Haase Jennifer, Hoff Eva V., Hanel Paul H. P., Innes-Ker Åse (2018). A meta-analysis of the relation between creative self-efficacy and different creativity measurements. Creativity Research Journal.

[B15-jintelligence-13-00025] Han Jiantao, Wang Yuwei, Qian Junni, Shi Menghua (2023). Delving into the role of creativity on meaning in life: A multiple mediation model. Heliyon.

[B16-jintelligence-13-00025] Holinger Molly, Kaufman James C. (2024). Measuring self-beliefs of creativity and well-being. Thinking Skills and Creativity.

[B17-jintelligence-13-00025] Jean-Berluche Ducel (2024). Creative expression and mental health. Journal of Creativity.

[B18-jintelligence-13-00025] Kashanian Amir Ashkan, Sheikhpour Mahmoud (2024). The Relationship Between Psychological Well-being and Creative Self-efficacy in High School Students. Journal of Adolescent and Youth Psychological Studies (JAYPS).

[B20-jintelligence-13-00025] Kaufman James C. (2012). Counting the muses: Development of the Kaufman domains of creativity scale (K-DOCS). Psychology of Aesthetics, Creativity, and the Arts.

[B21-jintelligence-13-00025] Kaufman James C. (2018). Finding meaning with creativity in the past, present, and future. Perspectives on Psychological Science.

[B22-jintelligence-13-00025] Kaufman James C. (2025). Dancing on an empty shore: Symbolic immortality, meaning, and being creative as doomsday approaches. Learning and Individual Differences.

[B19-jintelligence-13-00025] Kaufman James C, Beghetto Ronald A. (2009). Beyond big and little: The four c model of creativity. Review of General Psychology.

[B23-jintelligence-13-00025] Khumalo Itumeleng P., Wissing Marié P., Schutte Lusilda (2014). Presence of meaning and search for meaning as mediators between spirituality and psychological well-being in a South African sample. Journal of Psychology in Africa.

[B24-jintelligence-13-00025] King Laura A., Hicks Joshua A. (2021). The science of meaning in life. Annual Review of Psychology.

[B25-jintelligence-13-00025] Li Jian-Bin, Dou Kai, Liang Yue (2021). The relationship between presence of meaning, search for meaning, and subjective well-being: A three-level meta-analysis based on the meaning in life questionnaire. Journal of Happiness Studies.

[B26-jintelligence-13-00025] Li Yongzhan, Wu Dehui (2024). Creativity and Well-Being Among College Students: The Mediating Role of Meaning in Life. The Journal of Psychology.

[B27-jintelligence-13-00025] Love Jonathon, Selker Ravi, Marsman Maarten, Jamil Tahira, Dropmann Damian, Verhagen Josine, Ly Alexander, Gronau Quentin F., Šmíra Martin, Epskamp Sacha (2019). JASP: Graphical statistical software for common statistical designs. Journal of Statistical Software.

[B28-jintelligence-13-00025] Lyubomirsky Sonja, King Laura, Diener Ed (2005). The Benefits of Frequent Positive Affect: Does Happiness Lead to Success?. Psychological Bulletin.

[B29-jintelligence-13-00025] Mashkoor Maria, Muhammad Lakhi (2024). Being Happy Pays Off! Positive Affect Shaping Employee Creativity in the Hospitality Industry Through the Lens of Broaden-and-Build Theory. SAGE Open.

[B30-jintelligence-13-00025] McCrae Robert R. (1987). Creativity, divergent thinking, and openness to experience. Journal of Personality and Social Psychology.

[B31-jintelligence-13-00025] Newman David B., Nezlek John B., Thrash Todd M. (2018). The dynamics of searching for meaning and presence of meaning in daily life. Journal of Personality.

[B32-jintelligence-13-00025] Oishi Shigehiro, Westgate Erin C. (2021). A psychologically rich life: Beyond happiness and meaning. Psychological Review.

[B33-jintelligence-13-00025] Oishi Shigehiro, Choi Hyewon, Liu Ailin, Kurtz Jaime (2021). Experiences associated with psychological richness. European Journal of Personality.

[B34-jintelligence-13-00025] Oishi Shigehiro, Choi Hyewon, Koo Minkyung, Galinha Iolanda, Ishii Keiko, Komiya Asuka, Luhmann Maike, Scollon Christie, Shin Ji-eun, Lee Hwaryung (2020). Happiness, meaning, and psychological richness. Affective Science.

[B35-jintelligence-13-00025] Oishi Shigehiro, Choi Hyewon, Buttrick Nicholas, Heintzelman Samantha J., Kushlev Kostadin, Westgate Erin C., Tucker Jane, Ebersole Charles R., Axt Jordan, Gilbert Elizabeth (2019). The psychologically rich life questionnaire. Journal of Research in Personality.

[B36-jintelligence-13-00025] Peterson Christopher, Seligman Martin (2004). Character Strengths and Virtues: A Handbook of Classification.

[B37-jintelligence-13-00025] Richards Ruth (2018). Everyday Creativity and the Healthy Mind: Dynamic New Paths for Self and Society.

[B38-jintelligence-13-00025] Runco Mark A. (2007). A hierarchical framework for the study of creativity. New Horizons in Education.

[B39-jintelligence-13-00025] Ryff Carol D., Keyes Corey Lee M. (1995). The structure of psychological well-being revisited. Journal of Personality and Social Psychology.

[B40-jintelligence-13-00025] Silvia Paul J., Beaty Roger E., Nusbaum Emily C., Eddington Kari M., Levin-Aspenson Holly, Kwapil Thomas R. (2014). Everyday creativity in daily life: An experience-sampling study of ‘little c’ creativity. Psychology of Aesthetics, Creativity, and the Arts.

[B41-jintelligence-13-00025] Smith Kaile, Pickering Alan, Bhattacharya Joydeep (2022). The creative life: A daily diary study of creativity, affect, and well-being in creative individuals. Creativity Research Journal.

[B42-jintelligence-13-00025] Steger Michael F., Frazier Patricia, Oishi Shigehiro, Kaler Matthew (2006). The meaning in life questionnaire: Assessing the presence of and search for meaning in life. Journal of Counseling Psychology.

[B43-jintelligence-13-00025] Steger Michael F., Kashdan Todd B., Sullivan Brandon A., Lorentz Danielle (2008). Understanding the search for meaning in life: Personality, cognitive style, and the dynamic between seeking and experiencing meaning. Journal of Personality.

[B44-jintelligence-13-00025] Tan Chee-Seng, Lau Xiao-Shan, Kung Yian-Thin, Kailsan Renu A. (2019). Openness to experience enhances creativity: The mediating role of intrinsic motivation and the creative process engagement. The Journal of Creative Behavior.

[B45-jintelligence-13-00025] Tan Cher-Yi, Chuah Chun-Qian, Lee Shwu-Ting, Tan Chee-Seng (2021). Being creative makes you happier: The positive effect of creativity on subjective well-being. International Journal of Environmental Research and Public Health.

[B46-jintelligence-13-00025] Tang Min, Hofreiter Sebastian, Reiter-Palmon Roni, Bai Xinwen, Murugavel Vignesh (2021). Creativity as a means to well-being in times of COVID-19 pandemic: Results of a cross-cultural study. Frontiers in Psychology.

[B47-jintelligence-13-00025] Tierney Pamela, Farmer Steven M. (2002). Creative self-efficacy: Its potential antecedents and relationship to creative performance. Academy of Management Journal.

[B48-jintelligence-13-00025] Turiano Nicholas A., III Avron Spiro, Mroczek Daniel K. (2012). Openness to experience and mortality in men: Analysis of trait and facets. Journal of Aging and Health.

[B49-jintelligence-13-00025] Yu Wenjing, Qian Yiwei, Abbey Cody, Wang Huan, Rozelle Scott, Stoffel Lauren Ann, Dai Chenxu (2022). The role of self-esteem in the academic performance of rural students in China. International Journal of Environmental Research and Public Health.

